# Ultrafast nonlinear optical response in solution dispersions of black phosphorus

**DOI:** 10.1038/s41598-017-03667-z

**Published:** 2017-06-13

**Authors:** Lili Miao, Bingxin Shi, Jun Yi, Yaqin Jiang, Chujun Zhao, Shuangchun Wen

**Affiliations:** 1 Key Laboratory for Micro-/Nano- Optoelectronic Devices of Ministry of Education, School of Physics and Electronics, Hunan University, Changsha, 410082 China; 2 Femtosecond Optics Group, Department of Physics, Imperial College London, London, SW7 2BW UK; 3 Wuhan Optics Valley Aerospace Sanjiang Laser Industrial Technology Research Institute Co. Ltd., Wuhan, 430000 China; 4 Troop NO. 95829, Xiaogan, 432100 China

## Abstract

We report the spatial self-phase modulation (SSPM) effect for solution dispersions of black phosphorus (BP). The experimental results suggest that this concentration-dependent coherent light diffraction is due to the ultrafast and large third-order optical nonlinearity of BP. The third-order nonlinear susceptibility of BP has been simply obtained about 10^−19^ m^2^/V^2^ by analyzing the experimental results. The fast relaxation time during dynamic relaxation is obtained as 0.13 ps. Our experimental results imply novel potential application of BP in ultrafast nonlinear phase modulation devices based on their nonlinear optical response.

## Introduction

Low dimensional optical materials with ultrafast response and strong nonlinearity have exhibited novel physics and tremendous application potential in optical communication, optical sensing, medical diagnosis, etc^1–6^. Inspired by the emergence of two-dimensional atomic crystal graphene, the nonlinear optics of graphene and graphene-like materials have been explored intensively in recent years endowed for their excellent nonlinear response and broadband applications^7–10^. Besides graphene, black phosphorus (BP), a newly rising graphene-like material with natural band gap, has shown tremendous and intriguing physical, chemical, and electronic properties^11–13^. The BP has a thickness dependent direct band gap that changes from 0.3 eV in the bulk to 1.88 eV in a monolayer^14^. It can also be switched between insulating and conducting states, and it is still flat enough to confine electrons so that charge flows quickly, comparable with those in single layer of molybdenum disulphide^15^. Sample mobility is also found to be thickness dependent, with the highest value up to ~1000 cm^2^/Vs obtained at thickness ~10 nm^16^. Furthermore, the BP photodetector shows time response of 1 ms (rise) and 4 ms (fall), making few-layer BP a promising active 2D material in broadband and fast photodetectors across the visible and near infrared region^17^. In addition, BP is made from a single element, in theory, the pure samples are easier to obtain. Compared to other graphene-like 2D materials, tunable optical properties and direct bandgap for all thicknesses of BP may bring significant benefits to a variety of fascinating photonics applications.

Besides its high electron mobility, BP has unique optical properties, such as the strong in-plane anisotropic^18^. Considering the tunable band-gap, the electronic and optical performance of the materials can change dramatically consequently. Beyond the linear optics regime, the optical properties, especially the ultrafast optical response and the nonlinear optical properties of BP in case of different thicknesses, various doping conditions, and different polarization directions of the excitation light, have been paid more attention recently. The ultrafast relaxation dynamics of BP have been investigated by the pump-probe technique^19–21^, and the fast recovery time in BP was determined to be ~24 fs at 1550 nm^20^, 16 and 32 fs for 800 and 2026 nm pulses^21^, which is much faster than that of previous 2D crystal materials, such as graphene (~1.27 ps)^22^ and MoS_2_ (~2.1 ps)^23^. R. Suess *et al*. reported the anisotropic carrier dynamic with faster time to be ~180 ps at 780 nm in BP, which may be induced by hot carriers cooling^19^. In another aspect, the third-order nonlinear optical responses of BP have been investigated and characterized via Z-scan technique, third-harmonic generation (THG) method, etc^24–26^. The open-aperture (OA) Z-scan experiments demonstrate that BP has perfect saturable absorption performance at both optical communication band (1550 nm) and visible band (532 nm and 680 nm), making BP a preferred alternative material for saturable absorbers (SAs)^20^. The broadband nonlinear optics response of few-layer BP towards mid-infrared has also been reported by S. Lu *et al*.^24^. In addition, X. Zheng *et al*. reported a transition from saturable absorption (SA) to reverse saturable absorption (RSA) with the increase of laser intensity^26^. Moreover, both the passive Q-switching and the mode-locking operation of the erbium-doped fiber lasers with the BP-SA have been experimentally demonstrated^27^. D. Li *et al*. studied the thickness and polarization dependent linear and nonlinear optical properties of BP thin films, and then utilized their nonlinear absorption property to generate ultrafast and large-energy pulse with BP integrated fiber devices^28^.

Besides Z-scan and THG methods, spatial self-phase modulation (SSPM), a phenomenon that the intensity dependence of the refractive index in nonlinear optical media occurs, is a manifestation of the coherent response of the material. SSPM was originally observed in liquid-crystal^29, 30^, then later in nanomaterials, such as carbon nanotubes (CNTs) solutions^31^, graphene^32^, graphene oxide^33^, topological insulators^34^, transition metal dichalcogenides (TMDs)^35, 36^ and many other materials. While this manuscript was in preparation, a relevant work was published^37^. It reports the broadband spatial self-phase modulation of BP, and the nonlinear refractive index of BP (~5 layers) is measured to be ~10^−9^ m^2^/W and the third order nonlinear susceptibility is χ^(3)^ ~ 10^−8^ esu at multiple wavelengths (equals χ^(3)^ to be 10^−16^ m^2^/V^2^)^37^. Youngblood *et al*. report the χ^(3)^ of multilayer (~29 layers) BP to be 10^−19^ m^2^/V^2^ via layer tunable THG^25^, while Zheng *et al*. reported a nonlinear refractive index value of ~10^−13^ m^2^/W (~50–100 layers) at 800 nm femtosecond pulsed Z-Scan measurement^26^. In addition, from other perspective, these methods have given the different nonlinear optical parameters of BP^25, 26, 37^. It is exactly these disagreements that create the need for additional experimental and theoretical investigations to fully characterize the nonlinear susceptibility of BP further. In this work, we studied the phenomenon of SSPM for solution dispersions of BP under different concentration and laser intensity. The ultrafast nonlinear optical response was unambiguously observed and this coherent light diffraction is due to the remarkably large third-order nonlinearity. The third-order nonlinear refractive index of BP was then simply and exactly obtained by analyzing the experimental results.

## Results

### Characterizations of black phosphorus

**Figure 1:**
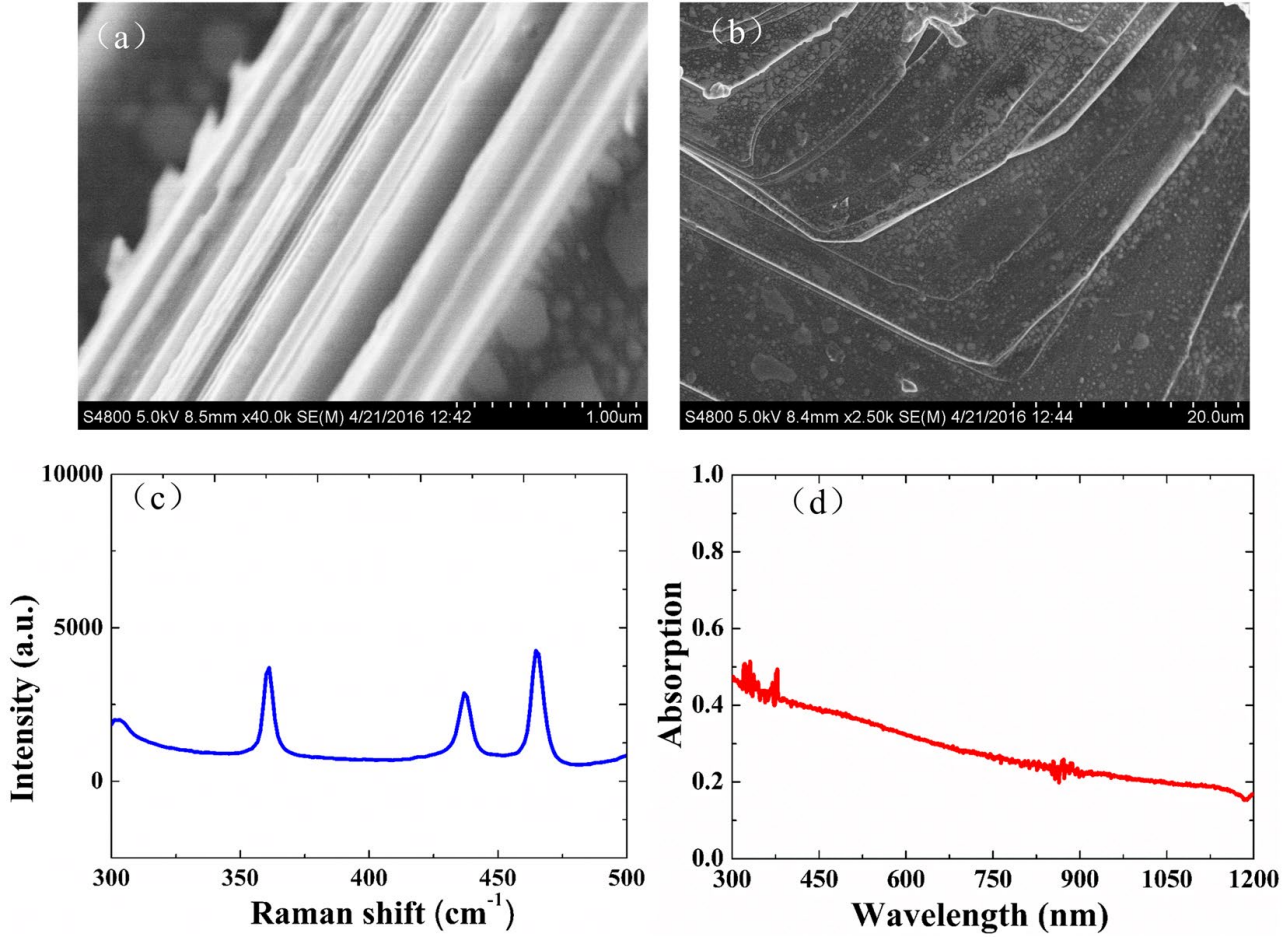
(**a**) and (**b**) SEM image of BP crystal, the scale bars are 1 μm (**a**) and 20 μm (**b**), respectively; (**c**) Raman spectra of BP; (**d**) Absorption spectrum of BP dispersion solutions in ethylene glycol.

Uniform few-layer BP dispersions have been prepared by the liquid phase exfoliation (LPE) method, which is a simple and effective technique to prepare two dimensional (2D) materials from layered bulk crystals towards multiple layered structures. Figure 1 shows the characterization of the BP sample. The scanning electron microscopy (SEM) image of BP crystal is shown in Fig. 1a,b, from which the high quality and flat layered surface of BPs can be verified from both lateral (a) and top (b) view, respectively. As shown in Fig. 1c, there are three Raman peaks at 361.15 cm^−1^, 436.85 cm^−1^, and 464.68 cm^−1^ from the multi-layer phosphorus corresponding to the ${{A}}_{{g}}^{1}$, ${B}_{2g}$, and $\,{A}_{g}^{2\,}$ modes, respectively. Figure 1d shows the linear absorption spectrum of the BP solution dispersions in ethylene glycol ranging from 300 to 1200 nm, measured by the spectrometer (Perkins Elmer Lambda750). The profile is nearly flat with a smooth absorption curve in the ultraviolet up to near-infrared (UV-NIR) wavelength band, suggesting that multi-layer BP dispersions might be a promising broadband optical material.

### Ultra-fast SSPM response

**Figure 2:**
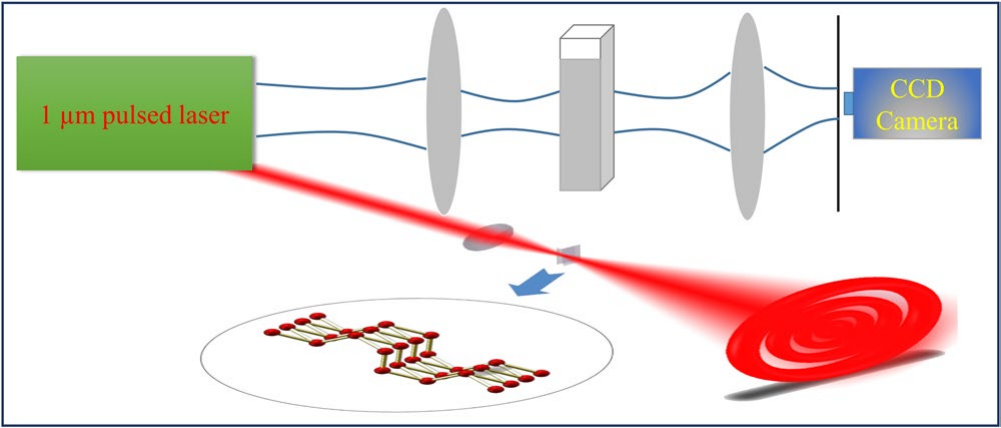
Experimental schematic diagram for SSPM experiment.

Inspired by the unique electronic and optical properties of BP, we have experimentally observed the SSPM phenomenon of BP. In the experiment, the ultrafast pulse laser was used to characterize the SSPM effects of BP solution dispersions. The linearly polarized beam from a 1061 nm (repetition rate of 2.7 MHz and pulse duration of 220 ps) laser was firstly focused by a lens of focal length 150 mm, causing high intensity illumination to the sample. A quartz cuvette of 5 mm thickness was used to contain the BP solution dispersions and placed before the beam focus. The distance between the focus and the front surface of the cuvette was 20 mm. Then the transmitted spectrum was collected by a CCD camera (Coherent LaserCam HR), which is 200 mm away from the focal lens. The schematic diagram was shown in Fig. 2.

**Figure 3:**
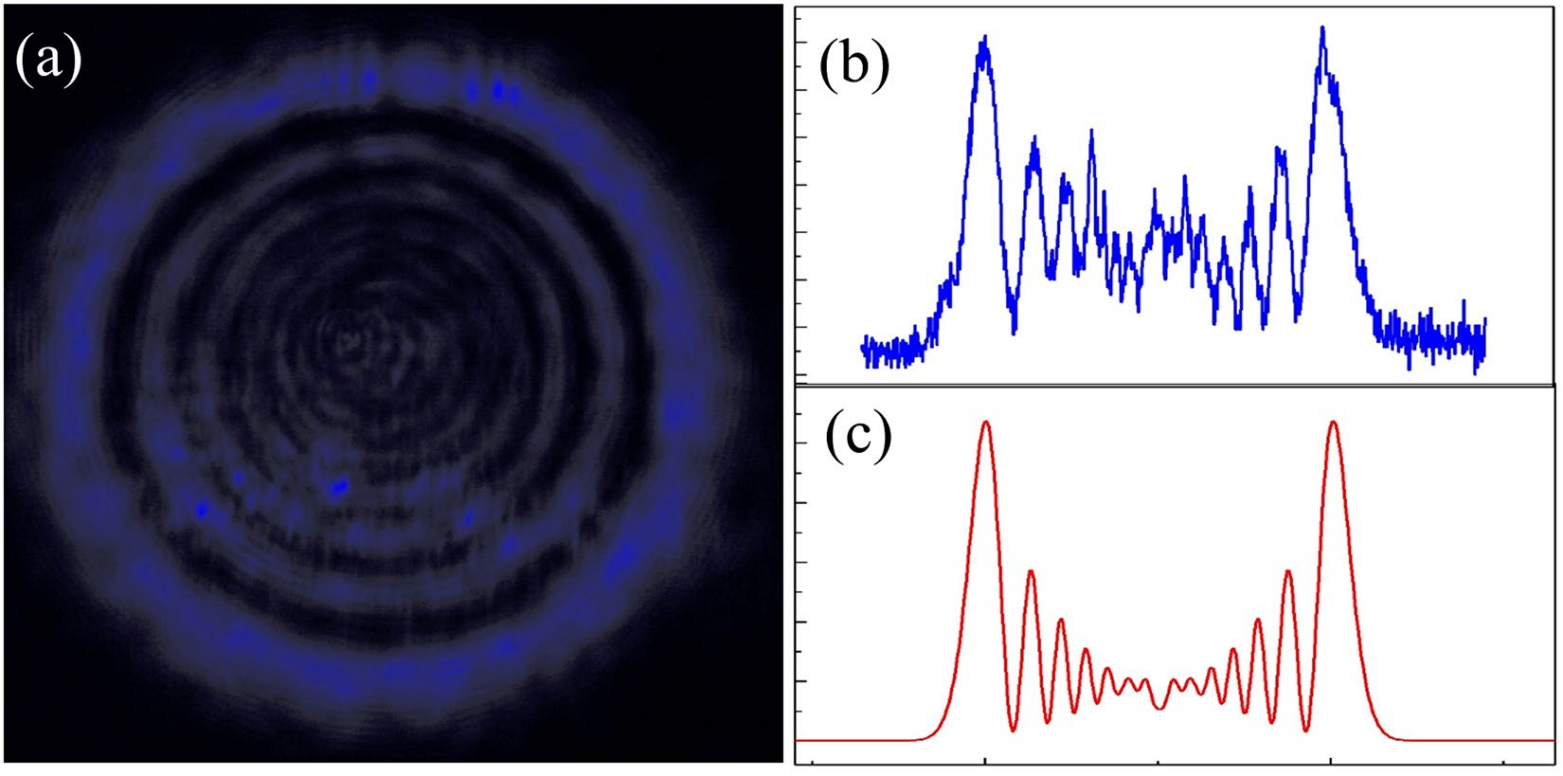
(**a**) The diffraction rings irradiated by 1061 nm ultrafast laser; (**b**) The axial intensity distribution of (**a**); and (**c**) The corresponding simulated intensity distribution.

Owing to the nonlinear index change induced by the nonuniform Gaussian profile of the light beam, we observed obvious diffraction ring pattern in the far field. Figure 3a is a typical pattern of diffraction rings caused by spatial self-phase modulation when the sample was irradiated by 1061 nm ultrafast laser. Figure 3b is the corresponding intensity distribution of the experimental result, and Fig. 3c illustrates the corresponding far field intensity distribution simulated by means of the Fraunhofer approximation of the Fresnel–Kirchhoff diffraction formula. From the figure, we conclude that the experimental result is in a good agreement with the theoretical one. Measurements of the pure ethylene glycol did not show a nonlinear response for the intensity ranges that were investigated here and, therefore, confirm that the observed nonlinear behavior is from the presence of the BP (*see supporting information*).

**Figure 4:**
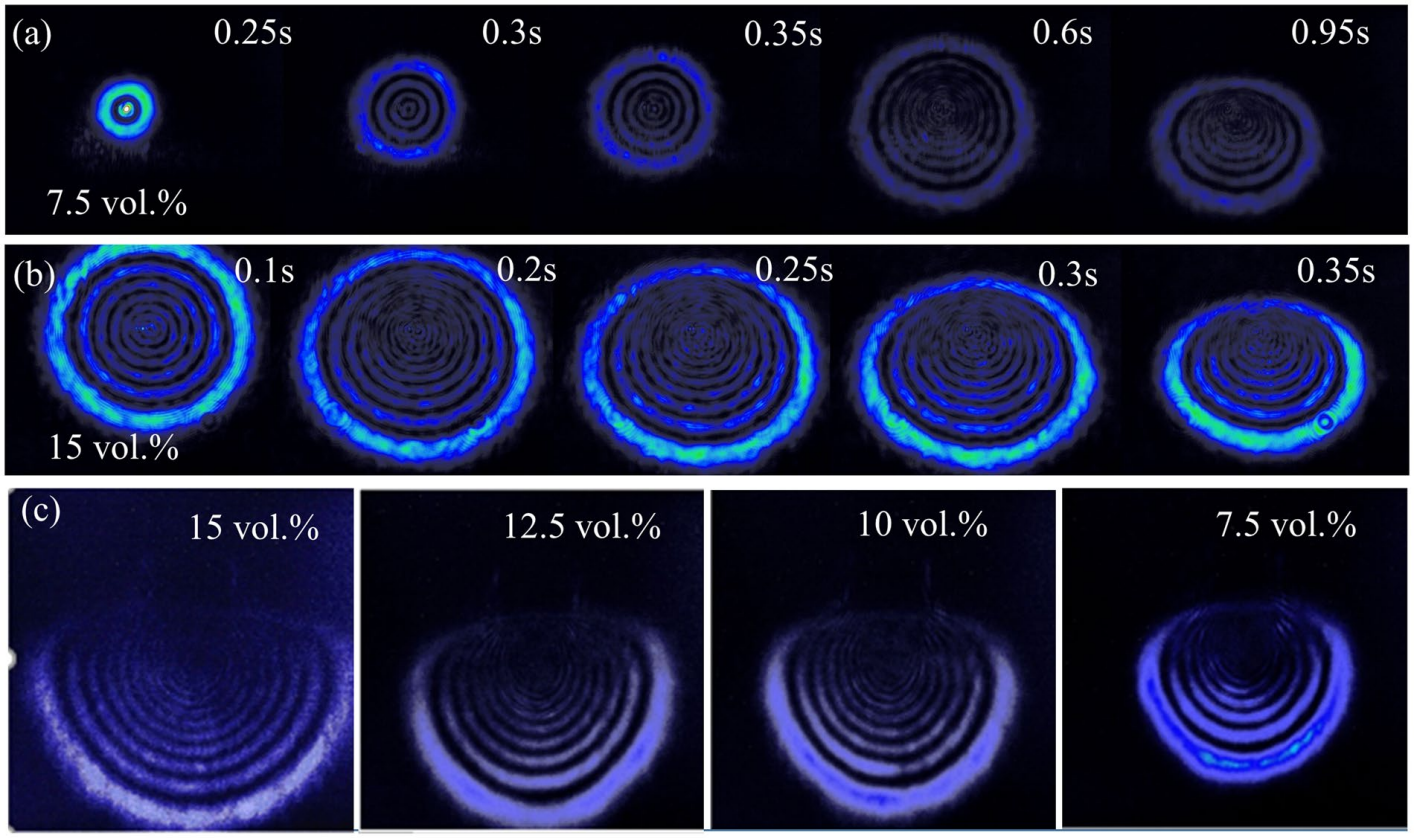
Snapshots of the pattern formation of: (**a**) Diluent BP solution dispersions (~7.5 vol.%); (**b**) 15 vol.% dispersion under the irradiance of 100 mW and (**c**) Different concentration under the irradiance of 140 mW at 1061 nm laser beam excitations.

To confirm the presence of the BP induces the nonlinear refractive index in the medium and causes the SSPM effect in laser beam propagation, we performed concentration-dependence measurements. Figure 4a,b show the typical development of diffraction rings of SSPM when the input average power is 100 mW. They both emerged from the center of the patterns with all their diameters enlarging gradually and they deformed with irradiation time evolution and finally become stable during one second. As shown in Fig. 4a, it takes about 0.95 second for the development of diffraction rings, which includes emerging, enlarging and deforming. While varying the concentration of the BP solution dispersion, the number of diffraction pattern increases, and the nonlinear response will become much faster with higher BP concentration. As the SSPM phenomena are proportional to the absorption of the nonlinear medium, pattern formation time, the size and number of rings become larger with the increasing of the concentration. Figure 4c shows the final stable patterns for four different concentrations of solution dispersions under the same input average power 140 mW, where the concentration decreases gradually from 15 vol.% to 7.5 vol.%, respectively. We can observe that both numbers and diameters of the diffraction rings decrease with the concentration. These results verify that diffraction rings are mainly derived from the nonlinear optical properties of BP.

**Figure 5:**
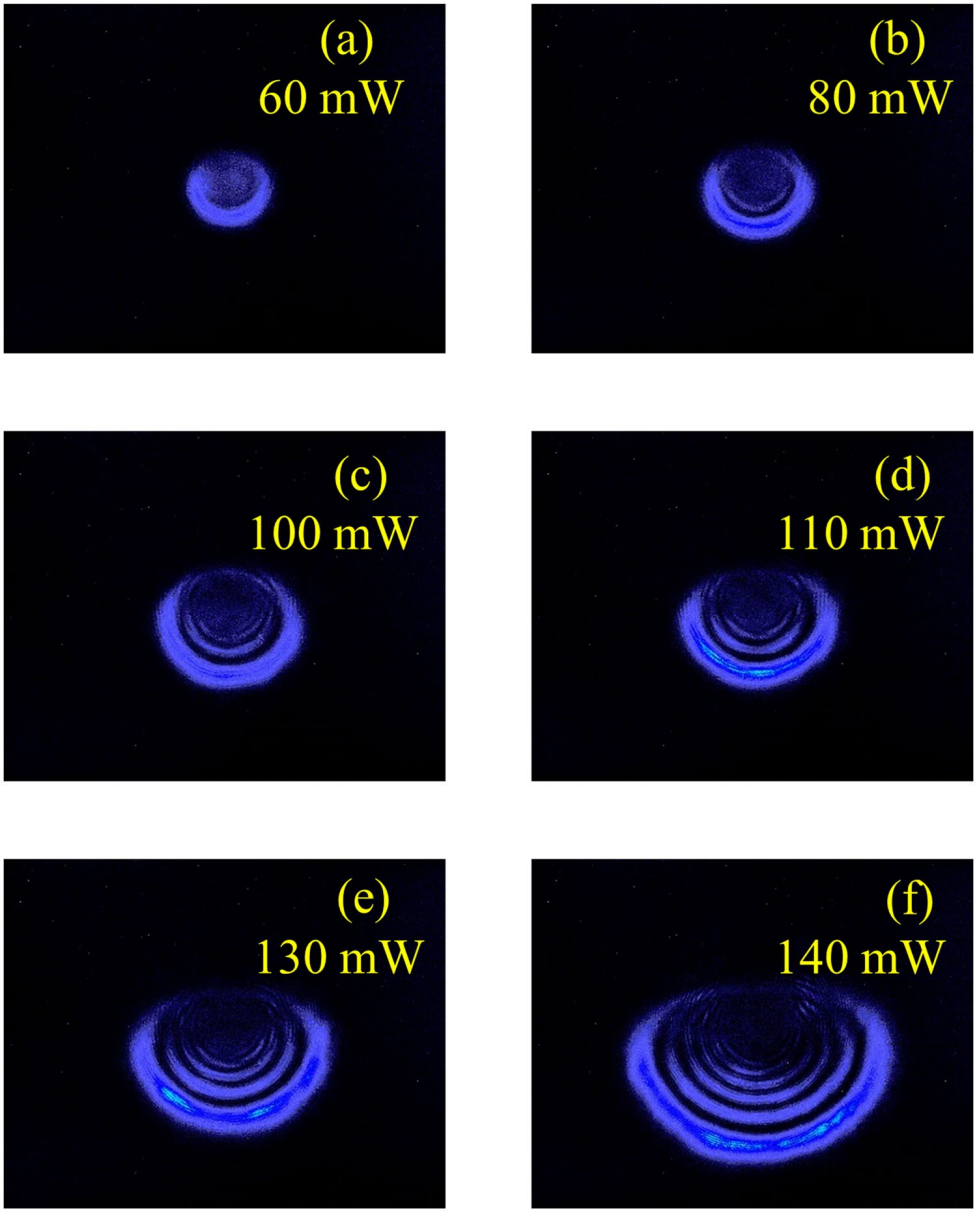
BP solution dispersions (~7.5 Vol.%) under input laser power increasing gradually from (**a**) to (**f**) as (**a**) 60, (**b**) 80, (**c**) 100 (**d**) 110, (**e**) 130 and (**f**) 140 mW, respectively.

We also performed intensity-dependence measurements with the same experimental setup to convince the nonlinear response of the BP dispersion. The input laser power increases gradually from Fig. 5a–f as 60, 80, 100, 110, 130 and 140 mW, respectively. It can be observed that by increasing the laser power, the number of rings and the vertical asymmetry of the diffraction ring patterns increased. Consequently, one can evidently confirm that when the input laser power becomes higher, the propagating beam suffers more serious distortions.

The refractive index of many materials can be described by the relation

1 $$n={n}_{0}+{n}_{2}I$$

where *n*_0_ is the linear refractive index and *I* is the laser intensity and *n*_2_ is the coefficient of the intensity-dependent refractive index^38^. The total refractive index increases with increasing optical intensity. A corresponding phase shift Δ*ψ* of the beam traversing the nonlinear medium^32^:

2 $${\rm{\Delta }}\phi (r)=\frac{2\pi {n}_{0}}{\lambda }{\int }_{0}^{Leff}{n}_{2}I(r,z)dz$$

where *r* is the radial coordinate of the beam, *λ* is the wavelength of incident laser, *L*_eff_ is the total propagation length contributing to the SSPM.

We assume, for simplicity, that

3 $${\rm{\Delta }}\phi (r)={\rm{\Delta }}\phi (0)\exp (\frac{-2{r}^{2}}{{\omega }^{2}})$$

where *ω* is the beam radius, as a constant. When $r\in [0,+\infty )$, there is *r*_1_ and *r*_2_ possessing the same wave vector and can interfere, which can be expressed as^29^:

4 $$\frac{d{\rm{\Delta }}\phi ({r}_{1})}{dr}=\frac{d{\rm{\Delta }}\phi ({r}_{2})}{dr}$$

The constructive or destructive interference occurs and results appearance of diffraction rings.

5 $${\rm{\Delta }}\phi ({r}_{1})-{\rm{\Delta }}\phi ({r}_{2})=m\pi $$

where *m* is an even or odd integer. The number of diffraction rings appeared can be estimated as^29^:

6 $$N=\frac{{\rm{\Delta }}\phi (0)-{\rm{\Delta }}\phi (r)}{2\pi }$$

Assuming Δ*ψ*(*r*) = 0, thus, $N={\rm{\Delta }}{\phi }_{0}/2\pi$, we can obtain the equation to solve *n*_2_^32^:

7 $${n}_{2}=(\frac{\lambda }{2{n}_{0}{L}_{eff}})\frac{N}{I}$$

**Figure 6:**
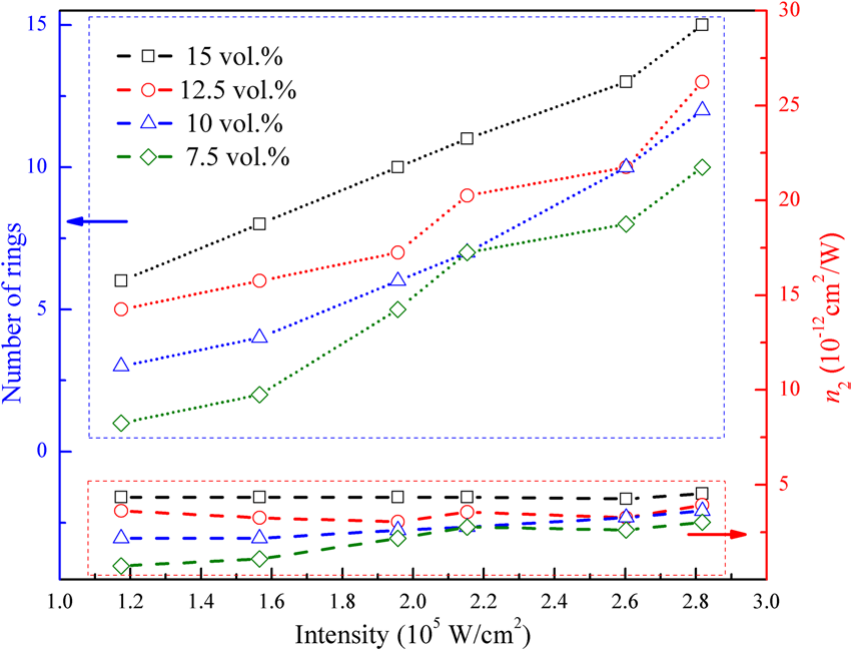
Relation between number of rings, nonlinear refractive index and input laser intensity.

The nonlinear refractive index and the third-order nonlinear susceptibility of BP at different concentrations have been summarized in Table 1. We compared the rings number, and nonlinear refractive index *n*_2_ at different concentration and input power which corresponds to different input intensity. At lower concentration, *n*_2_ increases with input power or intensity, while the saturation of *n*_2_ occurs due to sample concentration increase, as shown in Fig. 6. In our experiment, *N* is approximately proportional to *I* and the total refractive indexes increase with increasing optical intensity. The threshold for observing diffraction rings is about 0.58 × 10^5^ W/cm^2^ for 15 vol.% and 0.97 × 10^5^ W/cm^2^ for 7.5 vol.% BP solution dispersions. The nonlinearity *n*_2_ can be easily obtained by finding *N*/*I* at the maximum laser power. We can estimate *n*_2_ as 4.35 × 10^−12^ m^2^/W for 15 vol.% and 1.45 × 10^−12^ m^2^/W for 7.5 vol.% BP solution dispersions. The third order nonlinear susceptibility of BP was calculated to be 10^−14^ m^2^/ V^2^ (*see supporting information*). We use SI units here to avoid confusion. We estimate that ${\chi }_{total}^{(3)}={M}^{2}{\chi }_{{single}\,{layer}}^{(3)}$ ^32^. The number of effective layers *M* can be obtained as $M=\frac{C\times \,V\times \,{N}_{A}}{S/{a}^{2}}$, where *V* is the volume of the solution of 1.5 mL. The total number of (BP) molecules in the solution is *C* × *V* × *N*_A_, where *N*_A_ is the Avogadro’s number, *S* is the area of the cross-section of the cuvette ( = 1 cm × 3 cm), the total number of molecules in one effective layer is *S*/a^2^, where a (~4.5 Å) is the lattice constant of BP^39^. In our experiment, *M* is on the order of 300 to 600, thus the estimated ${\chi }_{single\,layer}^{(3)}$ is of the order of 10^−19^ m^2^/V^2^, which agrees with Z-scan measurements (~50–100 layers)^26^, two order of magnitude larger than estimated from THG experiments (~29 layers)^25^, but more than two orders of magnitude smaller than that from ref. 37 (~5 layers). The variation of nonlinear susceptibility by different methods can be attributed to the difference of BP thickness, as BP possesses a thickness-dependent energy band gap. Further efforts, from both theoretical and experimental sides, are needed to fully understand the third-order nonlinear optical response of BP, e.g. the relationship between formation time and BP concentration. The measurement uncertainty comes from several aspects: the calculation of incident pulse laser intensity, the measurement of distance between lens, sample, CCD camera, the concentration and uniformity uncertainty of the BP sample during the formation of diffraction ring, and the counting of diffraction rings number, which is a key parameter in calculating the nonlinear coefficient for BP. However, by well-designed experimental scheme and data analysis, SSPM phenomena can be regarded as an effective method to estimate the orders of magnitude of the nonlinear refractive index.

**Table 1:** The sample concentration and nonlinear optical parameters.

Volume fraction (vol.%)	*N*_ring number_	*n*_2_ (10^−12^ m^2^/W)	${{\boldsymbol{\chi }}}_{{\rm{t}}{\rm{o}}{\rm{t}}{\rm{a}}{\rm{l}}}^{(3)}$ (10^−14^ m^2^/V^2^)	*N*_layer number_	${{\boldsymbol{\chi }}}_{\mathrm{single}\,\mathrm{layer}}^{(3)}$ (10^−19^ m^2^/V^2^)
15	6	4.35	3.32	590	0.92
12.5	5	3.63	2.77	480	1.2
10	3	2.17	1.65	400	1.03
7.5	2	1.45	1.11	340	0.96

### Carrier dynamics

**Figure 7:**
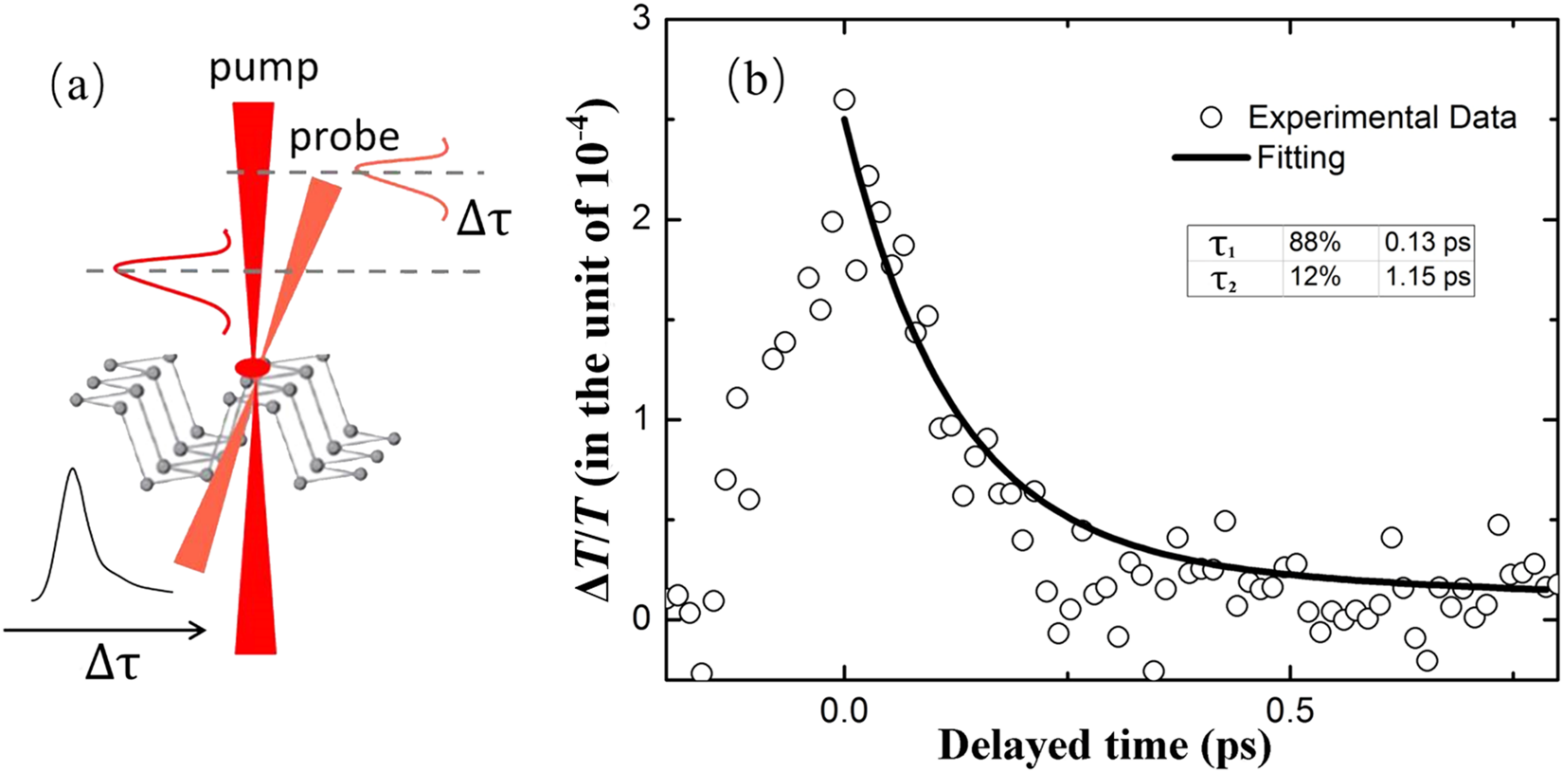
(**a**) Diagrammatic of carrier dynamics of BP solution; (**b**) Experimental results of pump-probe, scattered points are experimental results and solid lines are the fitting results.

The ultrafast nonlinear response of BP was also investigated by time-resolved optical degenerate pump-probe transmission measurement. Femtosecond (fs) laser (Coherent Libra-S), which gave an output with a pulse repetition rate of 1 kHz, a central wavelength of 800 nm and a pulse duration around 100 fs, was employed in the time-resolved pump-probe experiment. The output laser beam was split into two different laser beams, one intense portion with a peak intensity of 75 GW/cm^2^ as the pump beam to generate the photo-induced carriers, and the other weak portion with peak intensity of 0.3 GW/cm^2^ as the probe beam. Figure 7 presents the carriers dynamic relaxation process for the BP suspension. The delay kinetics can be well fitted with a bi-exponential delay function:

8 $$\frac{{\rm{\Delta }}T}{T}={A}_{1}{e}^{-t/{\tau }_{1}}+{A}_{2}{e}^{-t/{\tau }_{2}}$$

where Δ*T*/*T* is defined as the relative change of the probe transmissivity caused by the pump, and τ_1_ is the decay time with the respective amplitude weights *A*_1_. The fast and slow relaxation time is fitted to be 0.13 ps and 1.15 ps, respectively. We attribute the SSPM effect a transient phenomenon. The interaction and diffraction can occur under the well-defined phase difference between photons. To the ultrafast laser pulse, the carriers will relax in only about several pico-seconds, while every pulse is in the temporal slot of 0.37 μs (1061 nm, 2.7 MHz).

Therefore, the incident laser pulse is considered to be independent to each other, which confirms the transient effect of SSPM. The physical mechanism of the nonlinearity may be attributed to the reorientation and alignment of the BP nanosheets induced by the electromagnetic field, which is similar to the case of liquid crystals^29^. Under the intense laser irradiation, the polarization state will relocate to a new array which is parallel to each other. The reoriented sheets can help light beam become coherent and form SSPM rings.

## Conclusions

In conclusion, the third-order nonlinear susceptibility of BP dispersions was measured to be 10^−19^ m^2^/V^2^ and can be tuned via changing its concentration. Upon illumination, the BP solution dispersions show obvious SSPM response to the excitation wavelengths, demonstrating its ultrafast nonlinear response. Experimental results of BP under pulse illumination are fitted with a good correspondence between experimental and numerical results, which suggest that this coherent light scattering is due to the ultrafast, and large third-order optical nonlinearity of BP. The fast relaxation time during dynamic relaxation is obtained as 0.13 ps. Our experimental results exhibit the practical potential of this promising material for various nonlinear and ultrafast optoelectronics applications (e.g., ultrafast lasers, optical switches and modulators). Moreover, our work may provide an inroad for measuring key parameters, such as molecular weights of the solvent and the nanomaterials based on their novel nonlinear optical response. The technique can further be used to study the composition, purity and stability of other nanomaterials at broadband wavelength, including those with potential for clinical applications.

## Methods

### Materials

To begin, bulk BP crystal (purchased from Smart Elements) was grinded to BP powder. Then the powder was dispersed into ethylene glycol, along with ultra-sonicated for 2 hours. After sonication, the phosphorene in ethylene glycol was centrifuged at a speed of 15000 rpm for 20 minutes. The upper ~80% which contains few layers was decanted for analysis and the dispersions were settled for more than 20 hours to make sure that there is no large size sedimentations during our experiments. BP dispersions were then diluted to 4 samples (15 vol.%, 12.5 vol.%, 10 vol.%, 7.5 vol.%, respectively) for SSPM experiments.

## Electronic supplementary material

Ultrafast nonlinear optical response in solution dispersions of black phosphorus
