# Supplementary material for: Ultrafast nonlinear optical response in solution dispersions of black phosphorus
Source: Sci Rep. 2017 Jun 13;7:3352. doi: 10.1038/s41598-017-03667-z (PMC5469804; doi:10.1038/s41598-017-03667-z)
Supplement: Supplementary file 1 — Ultrafast nonlinear optical response in solution dispersions of black phosphorus [file 41598_2017_3667_MOESM1_ESM.pdf]

Ultrafast nonlinear optical response in solution dispersions of black phosphorus

Lili Miao[1,2], Bingxin Shi[3], Jun Yi[1], Yaqin Jiang[4], Chujun Zhao[1,*], and Shuangchun Wen[1]

[1]Key Laboratory for Micro-/Nano- Optoelectronic Devices of Ministry of Education, School of Physics and Electronics, Hunan University, Changsha 410082, China
[2]Femtosecond Optics Group, Department of Physics, Imperial College London, London SW7 2BW, UK
[3]Wuhan Optics Valley Aerospace Sanjiang Laser Industrial Technology Research Institute Co. Ltd., Wuhan 430000, China
[4]Troop NO.95829, Xiaogan 432100, China
Corresponding author, Email: chujunzhao@gmail.com

S1) Derivation of the Theoretical Simulation

The complex amplitude of the light electric field on the entrance plane of the medium can be written as

$$E(r,z_0)=E(0,z_0)\exp(-\frac{r^2}{w_p^2})\exp(-\frac{ik_0n_0r^2}{2R}) \tag{S1}$$

where w_p is the beam radius, k_0 is the free-space wavenumber, n_0 is the linear refractive index, R is the radius of wavefront curvature. When the Gaussian beam with the wavelength λ denoted by equation (1) propagated through the nonlinear medium, the transverse additional phase shift and the total phase shift induced on the exit plane of the medium can be expressed as

$$\Delta\phi(r)=k_0\int_{z_0}^{z_0+L}\Delta n(z,r)dz \tag{S2}$$

$$\phi(r)=\frac{k_0n_0r^2}{2R}+\Delta\phi(r) \tag{S3}$$

where L is the sample thickness. The complex amplitude of the electric field on the exit plane of the medium can be expressed as

$$\begin{aligned}E(r,z_0+L)&=E(0,z_0)\exp(-\frac{aL}{2})\exp(-\frac{r^2}{w_p^2})\exp(-i\phi(r))\\&\approx E(0,z_0)\exp(-\frac{aL}{2})\exp(-\frac{r^2}{w_p^2})\exp(-\frac{ik_0n_0r^2}{2R}-i\Delta\phi_0(z_0)\exp(-\frac{2r^2}{w_p^2})\end{aligned} \tag{S4}$$

When the optical field on the exit plane of the medium is freely propagated through the space, the corresponding far field intensity distribution can be expressed, by means of the Fraunhofer approximation of the Fresnel–Kirchhoff diffraction formula[1,2], as

$$I(D)=I_0\left|\int_0^\infty J_0(\frac{k_0\rho r}{D})\exp\left[-\frac{r^2}{w_p{}^2}-i\phi(r)\right]rdr\right|^2 \tag{S5}$$

where ρ is the far-field ring radius, D is the distance from the exit plane of the medium to the far-field observational plane, and J_0 is the first-kind zero-order Bessel function.

S2) Comparison of pure Ethylene Glycol

Measurements of the pure ethylene glycol was also conducted to confirm that the SSPM effect was induced by the presence of the BP. Figure S1 illustrates that pure ethylene glycol does not show a nonlinear response under input laser power of 150 mW, which is higher than the power we used for BP SSPM experiments.

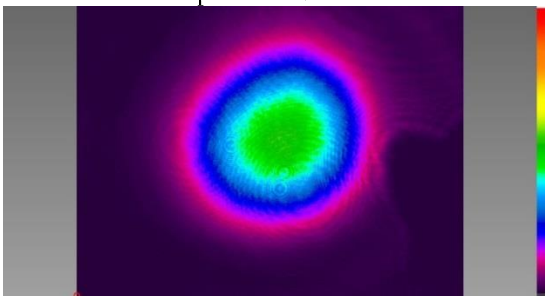

Figure S1. Transmitted beam spatial distribution captured by CCD of pure ethylene glycol by average power of 150 mW.

S3) Calculate χ^3 from n_2

The real part of the χ^3 can be obtained by the following equation[3]:

$$\mathrm{Re}\,\chi^3(esu) = \frac{c{n_0}^2}{120\pi^2} n_2(m^2/W) \tag{S6}$$

A conversion between the SI and CGS Systems of units can be carried out with the equation[4]:

$$\chi^3(m^2/V^2) = 1.4\times10^{-8}\,\chi^3(esu) \tag{S7}$$

References

1. Born, M and Wolf, E. Principles of Optics, Third Edition (Oxford: Pergamon, 1980).
2. Deng, L. G., He K., Zhou, T., and Li C., "Formation and evolution of far-field diffraction patterns of divergent and convergent Gaussian beams passing through self-focusing and self-defocusing media," *J. Opt. A: Pure Appl. Opt.* **7**, 409-415 (2005).
3. Zhang, W. F., Huang, Y. B., Zhang, M. S. and Liu, Z. G. Nonlinear optical absorption in undoped and cerium-doped $BaTiO_3$ thin films using Z-scan technique. *Appl. Phys. Lett.* 76 (2000).
4. Sutherland, R. L. Handbook of Nonlinear Optics, Second Edition (Marcel Dekker, New York, 2003).
